# A new species of *Parlatoria* from China (Hemiptera, Coccomorpha, Diaspididae)

**DOI:** 10.3897/zookeys.779.27437

**Published:** 2018-08-07

**Authors:** Minmin Niu, Jinian Feng

**Affiliations:** 1 Key Laboratory of Plant Protection Resources and Pest Management, Ministry of Education, Entomological Museum, College of Plant Protection, Northwest A&F University, Yangling, Shaanxi Province, 712100, China Northwest A&F University Yangling China

**Keywords:** A new species of armoured scale insect, *Parlatoriamenglaensis***sp. n.** is described and illustrated, which infests leaves of *Cinnamomumcamphora* in China. A key to the *Parlatoria* species occurring in China is provided. China, Diaspididae, Hemiptera, new species, *
Parlatoria
*

## Introduction

Armoured scale insects (Hemiptera: Coccomorpha: Diaspididae) are ubiquitous sap-sucking parasites that have a worldwide distribution (Andersen 2009). Compared with other family groups of Coccoidea, the armoured scale insects have several unique characteristics. For example, they have a cecum, which has no direct connection between the stomach and the anal opening, so they do not produce sweet secretions called honeydew (Henderson 2011). Females of the armoured scale insects are immobile and firmly attached to plant leaves, stems, fruits, or roots (Chou 1982). They have an extremely simplified morphology with several fused segments (Andersen 2010). They have no wings, or legs, and the eyes and antennae are reduced (Balachowsky 1948). The Diaspididae is the largest family of the Coccoidea with 2595 species currently identified (García Morales et al. 2018).

The genus, *Parlatoria* Targioni Tozzetti, 1868, is a large group of Diaspididae that are members of the tribe Parlatoriini. This genus was originally established by Curtis (1843) and *Aspidiotusproteus* Curtis, 1843, has been subsequently designated as the type species by Leonardi. The genus currently is made up of 73 species (García Morales et al. 2018). Some species of *Parlatoria*, such as *P.oleae* (Colvée) and *P.ziziphi* (Lucas) are considered to be serious pests of economic plants (McKenzie 1945).

*Parlatoria* is distributed in both tropical and subtropical regions (García Morales et al. 2018). This genus is found predominately in southeastern Asia but has also extended its range into Australia and Africa (McKenzie 1945). Some species have been introduced in the tropics and other warm parts of the world (Takagi 1969). About 34 species of this genus have been reported from China (García Morales et al. 2018).

Recently, a new species of *Parlatoria* was discovered in China and is described and illustrated in this study. This discovery raises the number of species recorded in this genus to 74, of which 35 are recorded from China. A key to the Chinese species of *Parlatoria* is presented in this study.

## Materials and methods

Plant samples infested by the new species were collected from Mengla city, Yunnan Province. Permanent slide mounts of adult females from the samples were prepared using the method described by Henderson (2011).

The illustrations of the adult female shown in Figs 1–9 were drawn from slide-mounted specimens. Fig. 1 shows an overview of the dorsal body surface on the left side and the ventral body surface on the right side, and an enlarged detail of the significant features of the body, which are not drawn in direct proportions to each other.

Slide-mounted type specimens of the new species have been deposited in the Entomological Museum, Northwest A&F University, Yangling, Shaanxi, China (NWAFU).

## Taxonomy

### Parlatoria

Taxon classificationAnimaliaBrassicalesBrassicaceae

Targioni Tozzetti

Parlatoria Targioni Tozzetti, 1868: 735.

#### Type species.

*Parlatoriaorbicularis* Targioni Tozzetti, subsequently designated by Leonardi (1899: 208).

#### Generic diagnosis.

**Female scale.** Various colors, oval to elongate, with exuviae terminal, often occupying greater part of scale.

**Male scale.** Elongate and smaller than the female.

**Adult female.** Female body nearly round or broadly ovate, and the widest at metathorax or abdominal segment I. The body free segments distinct but not strongly produced, and membranous except for the pygidium. Antennae with one or two setae. Prosomatic tubercles present or absent. Anterior spiracles with disc pores, the posterior spiracles without disc pores. Derm pocket present or absent between the posterior spiracle and body margin. Peribuccal granulations present or absent. The pygidium rounded or rather triangular. Three pairs of lobes well developed, unilobate, usually notched, median lobes not yoked; marginal macroducts of pygidium, one present or absent between the median lobes, one between the median and second lobes, and also between the second and third lobes. Two or three fimbriate plates present between lobes, then extend to the whole lateral margin of the pygidium, and each with one microduct. The fourth and fifth lobes often present, sclerotized or replaced by a membraneous fimbriate plates. Submarginal dorsal ducts usually present, scattered in a broad, continuous, irregular row on each side of the abdomen. Submedian dorsal ducts present or absent. Anal opening positioned about centre of pygidium. Four or five groups of perivulvar pores present (Adapted from McKenzie 1945, Takagi 1969, Williams and Watson 1988, Henderson 2011).

#### Remarks.

This genus *Parlatoria*, like other groups of the subfamily Aspidiotinae has an ovate body, and the second lobes are not divided into two lobules. Fringed plates are present between the lobes. *Parlatoria* is distinguished from other genera, especially *Parlagena* McKenzie, 1945, and *Parlatoreopsis* Lindinger, 1912, by having fringed plates across two sides of the prepygidial abdominal segments.

### Parlatoria menglaensis sp. n.

Taxon classificationAnimaliaBrassicalesBrassicaceae

http://zoobank.org/5F9869BC-25A3-4633-B445-6C6B76453DD8

Figures 1–9

#### Material examined.

Holotype and 30 paratypes, adult female. China: Yunnan Province. Mengla city, longitude 101.57, latitude: 21.48, on *Cinnamomumcamphora* (L.) Presl., 30.vii.2017, coll. Minmin Niu.

#### Description.

Female scale. Adult female cover convex, circular dark green/grey; exuvia on front end. Male scale. Not recorded.

**Adult female.** Body elongate-ovate, the broadest in first abdominal segment or thoracic region; segmentation distinct.

Antenna composed of two stout tubercles and a seta, located midway between frontal margin and mouthparts, interantennal distance being equal to width of mouth-parts. Prosomatic tubercles absent. Anterior spiracle with 1–2 parastigmatic pores; posterior spiracle without pores. Derm pocket absent between the posterior spiracle and body margin. Submarginal duct tubercules arranged as follows: 1–2 on cephaloprothoracic sternum, 3–5 on the mesothorax, and 4–8 on the metathorax.

Pygidium rounded, with 3 pairs of well-developed lobes. The median lobes distinctly notched once on each side, the second and third usually notched once distinctly on outer side. Fourth lobes replaced by a fimbriate plate, similar to adjacent fimbriate plates in form. Fimbriate plates present throughout pygidium, mostly as long as lobes, and two between median lobes, two between the median and second, three between the second and third; pygidial marginal macroducts largest closer to median lobes, arranged as follows: absent between the median lobes, one in each interlobar space, two outside the third lobe. Submarginal dorsal ducts on prepygidium numerous, 60–70 ducts on each side. Submedian ducts absent. Ventral microducts few, scattered on pygidium. Anal opening small, positioned about centre of pygidium. Perivulvar pores present in five groups; 3–4 in the median group, laterocephalic group with four pores, and the laterocaudal group with four pores.

**Figure 1–9.**
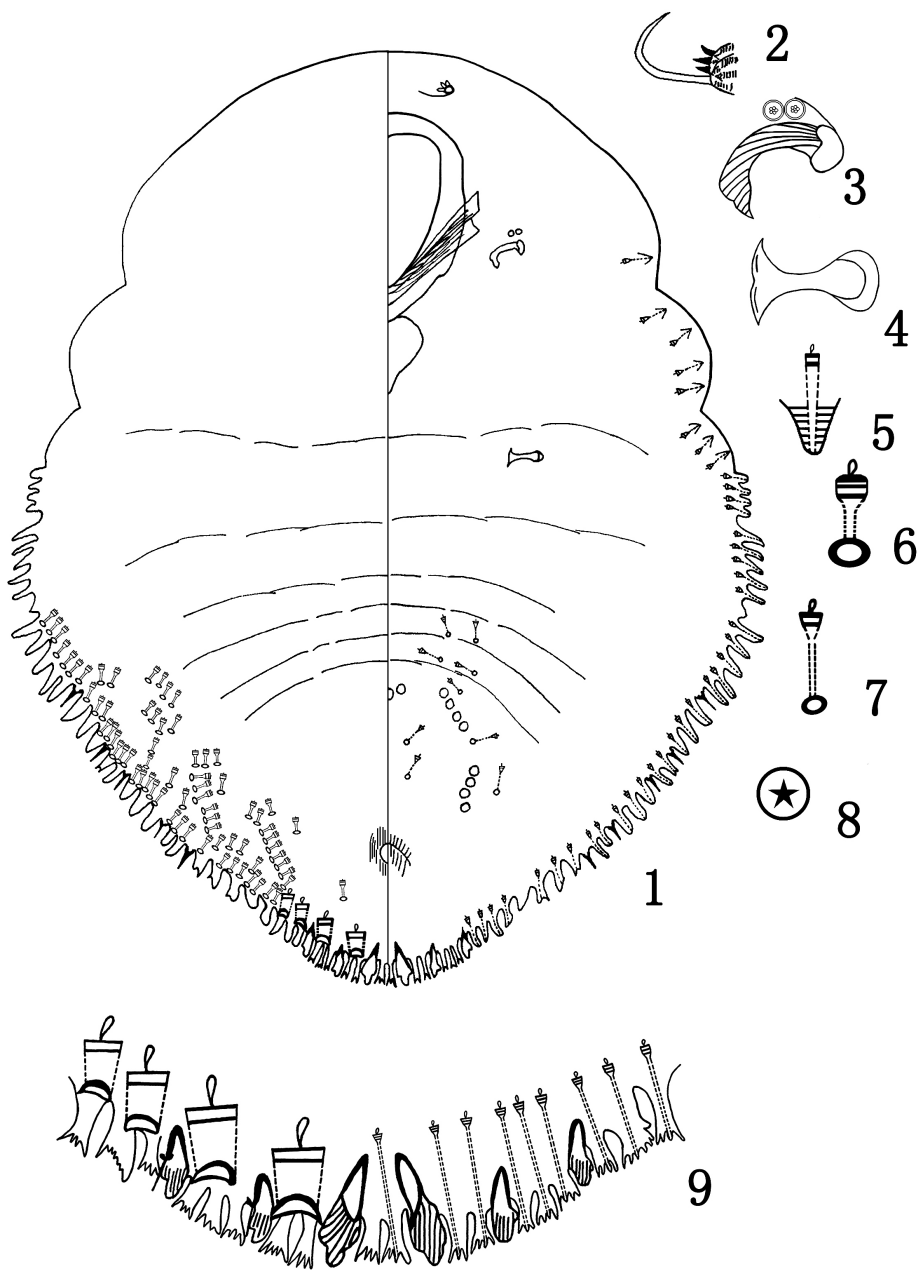
*Parlatoriamenglaensis* sp. n., adult female. **1** body **2** antenna **3** anterior spiracle **4** posterior spiracle **5** gland tubercle **6** detail of dorsal gland duct **7** ventral microduct **8** perivulvar pore **9** pygidium.

#### Remarks.

This species is very similar to *P.machilicola* (Takahashi 1933) in body shape, but differs in having (character-states on *P.machilicola* in brackets): (i) perivulvar pores present in five groups (perivulvar pores present in four groups); (ii) dorsal macroducts absent (dorsal macroducts present); (iii) marginal macroducts only four on each side (marginal macroducts more than four on each side).

The new species also resembles *P.tsujii* Tanaka, 2010, in the shape and spiracles. However, it differs from *P.tsujii* by the following characters (character-states on *P.machilicola* in brackets): (i) perivulvar pores present in five groups (perivulvar pores present in four groups); (ii) derm pocket absent (derm pocket present); (iii) marginal macroducts absent between the median lobes (marginal macroducts present between the median lobes).

#### Host plant.

*Cinnamomumcamphora* (L.) Presl (Lauraceae).

#### Etymology.

The specific epithet is named after Mengla, the type locality.

#### Distribution.

China (Yunnan).

##### Key to the adult females of *Parlatoria* Targioni Tozzetti from China

(There are records of *P.ligustri* Wu from China (Wu Chenfu 1935), but the information is inadequate for this key, thus it has not been included).

**Table:** 

1	Perivulvar pores absent	***P.pseudaspidiotus* (Lindinger)**
–	Perivulvar pores present in 4 or 5 groups	**2**
2	A marginal macroduct absent between the median lobes	**3**
–	A marginal macroduct present between the median lobes	**4**
3	Perivulvar pores absent in the median group	***P.machilicola* (Takahashi)**
–	Perivulvar pores present in the median group	***P.menglaensis* sp. n.**
4	Puparium of adult female black; with 1 peculiar, large and ear-like lobe on each side margin of head region about opposite anterior spiracles	***P.ziziphi* (Lucas)**
–	Puparium of adult female variable in color, but not black; on margin without ear-like lobes or lobes very small on each side of head region about opposite of anterior spiracles	**5**
5	Pygidium with 2 pairs of well-developed lobes, the 3^rd^ quite small, but sclerotized	***P.cupressi* (Ferris)**
–	Pygidium with 3 or more pairs of well-developed lobes	**6**
6	Anterior spiracles without disc pores	***P.mytilaspiformis* Green**
–	Anterior spiracles with disc pores	**7**
7	Derm pockets present between each posterior spiracle and body margin	**8**
–	Derm pockets absent between each posterior spiracle and body margin	**13**
8	Fourth lobes definitely present, not closely resembling adjacent plates	**9**
–	Fourth lobes absent, replaced by a membranous, plate-like process, smaller than the adjacent plates	***P.proteus* (Curtis)**
9	Four plates present between third lobe and fourth lobe	***P.pinicola* Tang**
–	Three plates present between third lobe and fourth lobe	**10**
10	Eyespots modified to form a stout spur	***P.crotonis* (Douglas)**
–	Eyespots various, flat, irregular or absent	**11**
11	Peribuccal granulations absent	***P.camelliae* (Comstock)**
–	Peribuccal granulations present	**12**
12	Pygidial lobes almost equal in size; perivulvar pores always in 4 groups	***P.pini* Tang**
–	Pygidial lobes of different sizes, the third pair smallest; perivulvar pores sometimes present in 5 groups	***P.theae* (Cockerell)**
13	Pygidium normally with four plates present between third lobe and fourth lobe	**14**
–	Pygidium with three plates present between third lobe and fourth lobe	**17**
14	Peribuccal granulations present	**15**
–	Peribuccal granulations absent	***P.multipora* McKenzie**
15	Dorsal ducts present on submedian area on abdominal segments I-III	***P.yanyuanensis* Tang**
–	Dorsal ducts absent from submedian area on abdominal segments I-III	**16**
16	Median lobes usually with a deep lateral notch and no medial notch (rarely with a small medial notch)	***P.oleae* (Colvée)**
–	Median lobes with deep lateral and medial notches	***P.bambusae* Tang**
17	Fourth lobes definitely present, not closely resembling adjacent plates	**18**
–	Fourth lobes replaced by a membranous, plate-like process, smaller than the adjacent plates	**27**
18	Lobes with outer margins normally notched	**19**
–	Lobes with outer margins minutely toothed	***P.yunnanensis* Ferris**
19	Lobes with outer margins with more than one notch	**20**
–	Lobes with outer margins with a single notch	**22**
20	Plates reduced	**21**
–	Plates well developed, fimbriate	***P.desolator* McKe nzie**
21	Dorsal median macroducts present on pygidium within frame formed by perivulvar pores	***P.cinerea* (Doane & Hadden)**
–	Dorsal median macroducts absent on pygidium within frame formed by perivulvar pores	***P.fluggeae* Hall**
22	Peribuccal granulations present	**23**
–	Peribuccal granulations absent	**24**
23	Submedian dorsal macroducts absent; Eyespot modified to form rounded elevation	***P.machili* Takahashi**
–	Submedian dorsal macroducts present on abdominal segments IV–V; Eyespot absent or inconspicuous	***P.cinnamomicola* Tang**
24	Median pygidial lobes deeply notched on outer and inner margins	**25**
–	Median pygidial lobes deeply notched only on outer margins	**26**
25	Perivulvar pores absent in the median group, antero- and posterolateral groups confluent on each side; submedian dorsal macroducts present on abdominal segments I-III	***P.emeiensis* Tang**
–	Perivulvar pores absent or a single one present in the median group, 6 to 8 in the anterolateral group, and 6 to 8 in the posterolateral group; submedian dorsal macroducts absent	***P.pergandii* (Comstock)**
26	Fourth lobes represented by small sclerotized spurs	***P.ghanii* Hall & Williams**
–	Fourth lobes well developed	***P.reedia* Zhang, Feng & Liu**
27	Peribuccal granulations absent	**28**
–	Peribuccal granulations present	**30**
28	Anterior spiracle with 1–3 disc pores	**29**
–	Anterior spiracle with 6–15 disc pores	***P.stigmadisculosa* Bellio**
29	Plates tapering apically to a point	***P.lithocarpi* Takahashi**
–	Plates well fimbriated	***P.acalcarata* McKenzie**
30	Perivulvar pores present in the median group, with 5 groups	***P.piniphila* Tang**
–	Perivulvar pores absent in the median group	**31**
31	Prosomatic tubercles rounded	***P.arengae* Takagi**
–	Prosomatic tubercles pointed apically, inconspicuous or absent	**32**
32	Posterolateral groups of perivulvar pores with 2–3 pores each	***P.hydnocarpus* Hu**
–	Posterolateral groups of perivulvar pores with more than 6 pores each	**33**
33	Gland tubercles: 1 to 3 prespiraculars, 3 to 4 anterior spiraculars, 3 to 5 mesothoracics, 4 metathoracics, and 4 first abdominals; submarginal dorsal ducts containing 18 to 22 on each side	***P.liriopicola* Tang**
–	Gland tubercles: 1 to 7 prespiraculars, 4 to 10 anterior spiraculars, 14 to 16 mesothoracics, 8 to 16 metathoracics, and 5 to 8 first abdominals; submarginal dorsal ducts containing 35 to 47 on each side	***P.keteleericola* Tang & Chu**

## Discussion

*Parlatoriamenglaensis* sp. n. is a pest in urban areas of China. Its primary host plant is *Cinnamomumcamphora* (L.) Presl (Lauraceae), which grows south of the Yangtze River in China. This plant is also found in southern Japan, Korea, and Vietnam, and has been introduced to many other countries, including Australia and the United States. Currently, *P.menglaensis* sp. n. has only been identified in China, but it could eventually spread to the previously mentioned countries where its potential host plants occur.

## Supplementary Material

XML Treatment for Parlatoria

XML Treatment for Parlatoria menglaensis
